# Mass spectrometry captures structural intermediates in protein fiber self-assembly†
†Electronic supplementary information (ESI) available: Supplementary methods and Fig. S1. See DOI: 10.1039/c7cc00307b



**DOI:** 10.1039/c7cc00307b

**Published:** 2017-02-01

**Authors:** Michael Landreh, Marlene Andersson, Erik G. Marklund, Qiupin Jia, Qing Meng, Jan Johansson, Carol V. Robinson, Anna Rising

**Affiliations:** a Department of Chemistry , Physical & Theoretical Chemistry Laboratory , University of Oxford , South Parks Road , Oxford , OX1 3QZ , UK . Email: Carol.Robinson@chem.ox.ac.uk; b Department of Anatomy , Physiology and Biochemistry , Swedish University of Agricultural Sciences , Uppsala , Sweden; c Department of Chemistry – BMC , Uppsala University , Box 576 , SE-751 23 , Uppsala , Sweden; d Institute of Biological Sciences and Biotechnology , Donghua University , Shanghai , 201620 , P. R. China; e Division of Neurogeriatrics , Department of Neurobiology , Care Sciences and Society (NVS) , Center for Alzheimer Research , Karolinska Institutet , Huddinge , 14157 , Stockholm , Sweden . Email: Janne.Johansson@ki.se ; Email: Anna.Rising@ki.se

## Abstract

Self-assembling proteins, the basis for a broad range of biological scaffolds, are challenging to study using most structural biology approaches. Here we show that mass spectrometry (MS) in combination with MD simulations captures structural features of short-lived oligomeric intermediates in spider silk formation, providing direct insights into its complex assembly process.

## Abstract 2

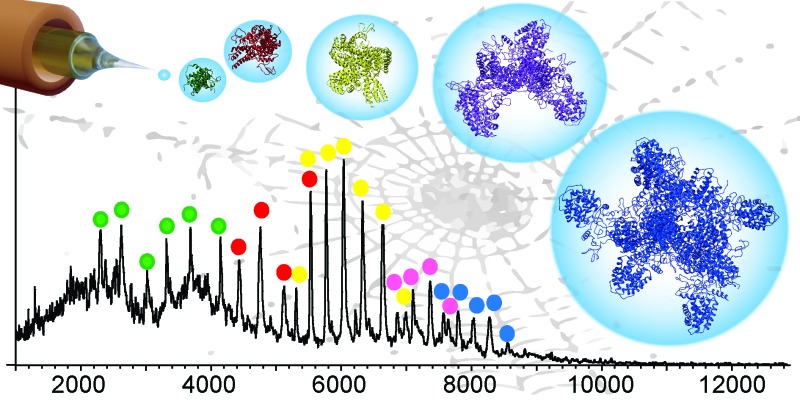
Integrating ion mobility mass spectrometry and molecular dynamics simulations provides insights into intermediates in spider silk formation. The resulting structural models reveal how soluble spidroin proteins use their terminal domains to assemble into silk fibers.

## 

No protein assembly matches the combined strength and extensibility of spider silk, consequently it bears great promise for biomedical applications.^1,2^ Its primary constituents are spidroins, proteins with a highly repetitive amino acid sequence flanked by small N- and C-terminal non-repetitive domains. The structure of the silk fiber consists of poly-alanine stretches in the repetitive regions^3^ which form crystalline β-blocks that are connected by partially disordered segments with high glycine content. The exceptional properties of the silk are attributed to this unique architecture.^4,5^ The processes that govern the assembly of these spidroins into silks remain a challenge for study using established structural biology tools. In their soluble state, the flexibility of the spidroins precludes crystallization, while their size hampers detailed NMR studies. In addition, their assembly is near instantaneous and largely outside of the time frames afforded by either technique. Yet, understanding the molecular basis of the self-assembly of spidroins and their regulation is crucial for harnessing the remarkable properties of the silk. We therefore investigated whether non-denaturing MS is able to capture previously inaccessible features of the spidroin assembly.

Spidroins are stored in the spider's silk gland as a fluid dope at concentrations exceeding 30% w/v.^6^ During spinning, the dope undergoes a pH drop from 7.6 to <5.7 with increasing CO_2_ and changes in salt concentrations, and is exposed to shear force by being pulled through a narrowing duct.^7^ Native spidroins contain many aggregation-prone repeats and have a molecular weight of up to 300 kDa, which makes them refractory to detailed structural analysis. Functional studies rely on insights from isolated domains: the pH decrease causes the dimerization of N-terminal domains (NT),^8–12^ whereas the C-terminal domain (CT) is destabilized.^7,13,14^

While differences in the repeat domains determine silk properties, nearly all types of spider silk contain homologues of both domains.^8,14–16^ How these structural changes together control the assembly of repetitive regions, over 30 times their own size, remains an intriguing question. To circumvent the difficulties in handling the extremely unstable native spidroins, we designed a mini-spidroin (NT2RepCT) with a shortened repeat domain (Fig. 1A) composed of the N-terminal domain and two ensemble repeats from the *Euprosthenops australis* major ampullate spidroin 1 (MaSp1) and the C-terminal domain from the *Araneus ventricosus* minor ampullate spidroin 1 (MiSp1).

**Fig. 1:**
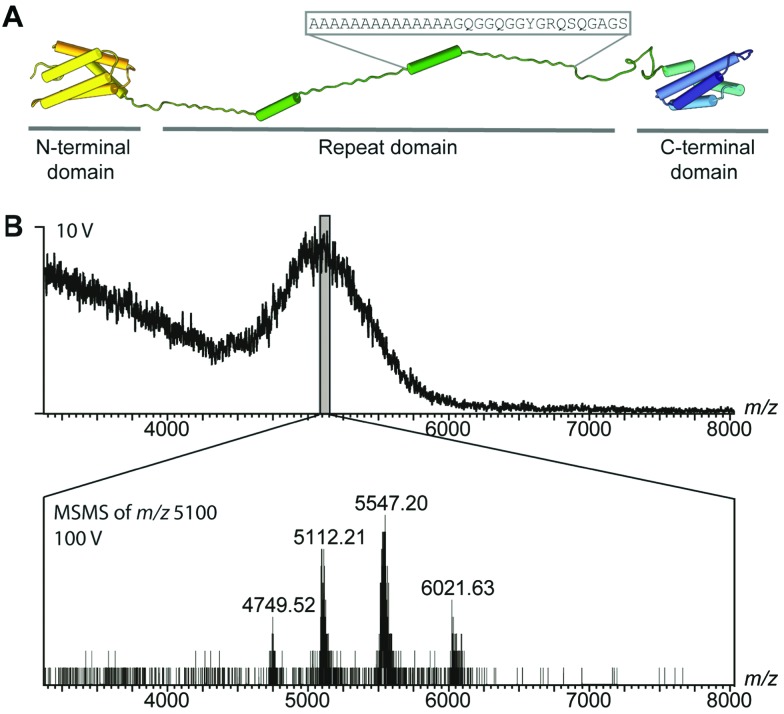
MS of the mini-spidroin NT2RepCT. (A) NT2RepCT contains the N-terminal domain (yellow) and two repeats (green) from *E. australis* MaSp1 (4FBS), fused to the C-terminal domain (blue) of MiSp1 from *A. ventricosus* (2MFZ). The sequence of one repeat is indicated above the structure. (B) nESI-MS spectra of NT2RepCT diluted in ammonium acetate, pH 7.5, at a collision voltage of 10 V show unresolved ensembles. Isolation of *m*/*z* 5100 and tandem MS reveal a single charge state series of NT2RepCT dimers.

The terminal domains were chosen for their well-established pH-dependent behavior and a high degree of similarity to other spidroin terminal domains. NT2RepCT is soluble at pH 7.5 and electron microscopy (EM) shows the presence of spherical assemblies 7–9 nm in diameter.^17^ Similar structures have been observed for other spidroins and are believed to represent the storage form in the silk gland.^18,19^ The exposure of concentrated NT2RepCT to pH 5.5 and simultaneous shear force readily generate fibers with silk-like properties.^17^ To investigate the structure and dynamics of NT2RepCT under assembly conditions, we used mass spectrometry (MS) in combination with ion mobility spectrometry (IM). MS is well suited to monitor the stoichiometry of individual populations of protein complexes, while IM–MS is able to assess their overall conformation, as recently demonstrated for viral capsid assembly.^20,21^ First, we investigated the oligomeric state of soluble mini-spidroins. The nano-electrospray ionization mass spectrometry (nESI-MS) of NT2RepCT stock solutions diluted with ammonium acetate, pH 7.5, shows a broad, unresolved peak (Fig. 1B). Since its appearance suggests the presence of heterogeneous oligomeric states and multiple adducts, we employed collisional activation in the ion trap of the mass spectrometer to investigate their composition.^22^ Upon selective activation the ensembles dissociated completely into a single molecular species of 65 kDa, corresponding to non-covalent NT2RepCT dimers, in good agreement with studies on isolated domains that show a monomeric N-terminal and a dimeric C-terminal domain at pH 7.5.^9,10,12,14,16,23^ When a gel filtration step was included immediately before MS analysis, we observed intact dimers in the mass spectra recorded at a low collision voltage (10 V) (Fig. 2A).^17^ At higher collision voltage (100 V), we observed predominantly NT2RepCT dimers as well as a small population of tetramers (Fig. 2A lower). Together, these observations suggest that at pH 7.5 NT2RepCT forms dimers that loosely associate into small oligomers.

**Fig. 2:**
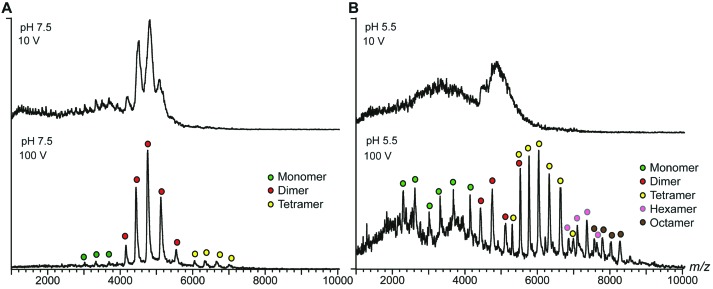
nESI-MS shows pH-dependent spidroin polymerization. (A) nESI-MS of NT2RepCT at pH 7.5 after gel filtration shows well-resolved dimers. Increasing the collision energy improves spectral resolution to show minor monomer and tetramer populations. (B) Acidification of the sample in the electrospray capillary by direct addition of dilute formic acid results in a significant loss of signal intensity. Simultaneous collisional activation reveals a shift towards higher oligomeric states of NT2RepCT, which are centered on tetramers but span from monomers to octamers.

Next, we investigated how pH changes affect the oligomeric status of NT2RepCT. However, adjusting the pH to 5.5 before MS analysis resulted in a complete loss of the NT2RepCT signal.^17^ To overcome this problem, we lowered the pH through step-wise addition of dilute formic acid directly in the electrospray capillary. At a final formic acid concentration of 0.02%, we observed a sudden loss of signal intensity (Fig. 2B, upper panel). However, collisional activation revealed well-defined peaks that show a significant shift to higher oligomers of NT2RepCT (Fig. 2B, lower panel). The assignment of these oligomers reveals predominantly tetramers with hexamers and octamers and demonstrates a clear preference for even-number subunit stoichiometries (Fig. 2B and Fig. S1, ESI†). Subjecting the oligomers to collisional activation releases tetramers, hexamers and octamers of NT2RepCT with a relative abundance that decreases with the oligomer size. Using an Orbitrap mass spectrometer modified for high-mass analysis,^24^ we could additionally detect decamers (Fig. S2, ESI†). Oligomers could only be observed immediately after lowering the pH, and the signal intensity was found to decline rapidly. Our findings can be rationalized with the proposed roles of the terminal domains: the dimeric C-terminal domain aggregates at low pH while the N-terminal domain undergoes rapid dimerization, in line with the observation that lowering the pH triggers near-instant polymerization of NT2repCT dimers followed by a rapid loss of soluble protein.

To further characterize the structures of the assembly intermediates, we analyzed the different oligomeric states using IM–MS. Briefly, desolvated protein molecules are injected into a gas-filled cell and separated according to their charge and collision cross-section (CCS). These properties are manifested in their drift times and reflect their respective folding states. In this manner, IM–MS yields information about protein conformations in the gas phase. A comparison of the CCS of monomeric and oligomeric NT2RepCT to the CCS of an idealized spherical protein reveals that the conformations of all oligomeric states agree well with the cross-sections expected for globular assemblies of the same molecular weight (Table S1 (ESI†) and Fig. 3A). The CCS values of the isolated NT and CT dimers, for which similar correlations were observed, are in good agreement with the theoretical values calculated from their respective high-resolution structures (Table S1, ESI†). Collision-induced unfolding of the NT2RepCT dimer reveals a step-wise unfolding profile indicative of a soluble multi-domain protein (Fig. 3C).

**Fig. 3:**
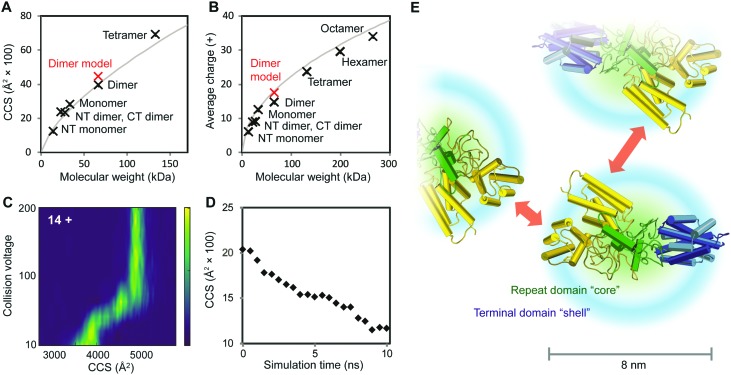
Structural features of intermediates in the spidroin assembly. (A) At pH 7.5, the CCS values of CT and NT as well as monomeric, dimeric and tetrameric NT2RepCT correspond to those expected for globular proteins. The theoretical CCS of a spherical protein with a density of 0.69 g cm^–3^ is shown in grey. (B) Analysis of the charge states of NT, CT, and NT2RepCT oligomers observed at pH 5.5 shows a trend towards compact conformations. The expected average charge of a spherical protein with a density of 0.69 g cm^–3^ is shown in grey. (C) Collision-induced unfolding of the 14+ charge state of the NT2RepCT dimer at pH 7.5 gives rise to a step-wise unfolding profile indicative of a folded protein. (D) MD simulations of the repeat domain reveal rapid compaction starting from the extended conformation shown in Fig. 1. (E) The structural model of an intermediate in the silk assembly. Isolated NT2RepCT dimers adopt a compact structure with a condensed repeat domain “core” and solvent-exposed terminal domains. This structure makes the N-terminal domain (yellow) accessible for antiparallel dimerization, which mediates the formation of globular polymers. The theoretical CCS value and predicted average charge of the NT2RepCT dimer model are shown as red markers in (A) and (B). Domains are colored as in Fig. 1A.

As the net charge of a desolvated protein is empirically related to its surface area, the observed charges can be compared to the charges expected for a globular protein of the same molecular weight.^25^ Strikingly, the charge states of the desolvated polymers formed at low pH also correspond to those expected for a compact folded protein (Fig. 3B). This suggests that the pH-induced polymerization of NT2RepCT dimers does not lead to significant deviations from their globular conformation.

To relate the information from the IM–MS measurements to the conformational states in solution, we performed MD simulations of the repeat domain, which is the flexible part of the protein. Monitoring the theoretical CCS over the course of the trajectory reveals that the mixed helix/random coil repeats readily adopt a compact conformation (Fig. 3D). We also observe that the RMSD values across the trajectory show a rapid convergence towards the final structure of the simulation, indicating that the repeat domain adopts a single preferred conformation (Fig. S3, ESI†). The resulting structural model (Fig. 3E) agrees with the CCS values and charge states that we determined for the intact spidroin dimers (Fig. 3).

The finding that NT2RepCT retains its compact conformation in MS may appear surprising. Proteins with large unstructured segments commonly exhibit charge states and CCS values indicative of a co-existence of compact and extended conformations.^26^ With increasing length, however, the unstructured protein segments acquire a tendency to populate globule-like states.^27^ The charge states and CCS values measured for NT2RepCT during the assembly show that it forms polymers composed of compact dimers. DLS and NMR studies have suggested that recombinant spidroins lacking the N-terminal domains preferentially adopt similar conformations in solution independent of pH.^28,29^ The diameter distribution of 7–9 nm observed by EM for NT2RepCT oligomers corresponds to a molecular weight of 200–350 kDa assuming an average density of 0.69 g cm^–3^. The corresponding oligomers would therefore be composed of six to twelve NT2RepCT monomers, in reasonable agreement with the largest polymers (8–10 protomer units) that can be detected by MS.

In conclusion, the insights from the MS and MD capture several stages of the silk assembly process: the soluble spidroins form compact, non-covalent dimers and conceal their aggregation-prone repeat domains by adopting globular conformations in which the terminal domains are exposed. pH-Induced rapid polymerization of the spidroins yields amorphous oligomers that can be spun into silk through simultaneous exposure of the concentrated spidroin solution to shear force.^17,30^ We anticipate that the approach employed here is amendable to a wide variety of biomaterials.

ML holds an ERC Marie Curie Early Career Development Grant and is a Junior Research Fellow of St Cross College, University of Oxford. EGM holds a Marie Skłodowska Curie International Career Grant from the European Commission and the Swedish Research Council, and is a Fulford Junior Research Fellow at Somerville College, University of Oxford. JJ is supported by the Swedish Research Council (2014-10371) and Center for Innovative Medicine (CIMED) at Karolinska Institutet and Stockholm City Council. CVR is supported by an ERC Advanced Investigator Award ENABLE (641317), a Welcome Trust Investigator Award (104633/Z/14/Z), and a Medical Research Council Program Grant (MR/N020413/1). AR is supported by FORMAS (942-2015-629) and the Swedish Research Council (2014-2408).
