# Supplementary material for: Mass spectrometry captures structural intermediates in protein fiber self-assembly
Source: Chem Commun (Camb). 2017 Feb 1;53(23):3319–22. doi: 10.1039/c7cc00307b (PMC5530726; doi:10.1039/c7cc00307b)
Supplement: Supplementary file 1 [file CC-053-C7CC00307B-s001.pdf]

Electronic Supplementary Material (ESI) for ChemComm.
This journal is © The Royal Society of Chemistry 2017

Supplementary Information for

Mass spectrometry captures structural intermediates in protein fiber self-assembly

Michael Landreh, Marlene Andersson, Erik G. Marklund, Qiupin Jia, Qing Meng, Jan Johansson, Carol V. Robinson, Anna Rising

Experimental Procedures

NT2RepCT (MW 32509 Da, Sequence: MGHHHHHHMSHTTPWTNPGLAENFMNSFMQGLSSMPGFTASQLDDMSTIAQSMVQSIQSLAAQGRTSPNKLQALNMAFASSMAEIAASEEGGGSLSTKTSSIASAMSNAFLQTTGVVNQPFINEITQLVSMFAQAGMNDVSAGNSGRGQGGYGQGSGGNAAAAAAAAAAAGYGRQSQGAGSAAAAAAAAAAAAAAGSGQGGYGGQGQGGYGQSGNSVTSGGYGYGTSAAAGAGVAAGSYAGAVNRLSSAEAASRVSSNIAAIASGGASALPSVISNIYSGVVASGVSSNEALIQALLELLSALVHVLSSASIGNVSSVGVDSTLNVVQDSVGQYVG, as well as isolated NT and CT (the corresponding sequences are underlined) were expressed as described previously and stored in 20 mM Tris buffer, pH 8.0 [1, 2] Proteins were either diluted 10-fold in 100 mM ammonium acetate, pH 7.5 from the ~100 µM stock solution, or subjected to gel filtration into 100 mM ammonium acetate, pH 7.5 using biospin columns (Bio-Rad Laboratories) and analysed immediately. Samples were introduced into the mass spectrometer using gold-coated borosilicate capillaries produced in-house. To lower the pH, samples were loaded into a capillary and a spectrum was recorded. The capillary was then removed from the holder, 0.1% formic acid was added to a final concentration of ~0.02% using a gel-loader pipette tip, and the capillary was immediately placed back and another spectrum recorded. A constant desolvation gas flow of 0.2 mL/h N_2 was required to extrude the low pH sample. Spectra were recorded on a modified high-mass Q-Exactive Orbitrap mass spectrometer (Thermo),[3] or a Synapt G1 T-wave ion mobility mass spectrometer (Waters). The Orbitrap settings were: Capillary voltage, 1.4 kV; HCD collision energy 200 V; HCD cell pressure 1×10^{-9} mbar; collision gas, argon. The Synapt settings were: capillary voltage, 1.5 kV; sample cone 40 V; source temperature, 20 °C; cone gas, off; trap collision energy, 10 V or 100 V as indicated; transfer collision energy, 10 V; trap DC bias 8 V; backing pressure 6.8 mbar; trap gas was argon with a flow of 8 mL/min; IM gas was helium with a flow of 20 mL/min; IMS wave velocity 250 m/s; IMS wave height, 12 V; transfer wave velocity, 248 m/s, transfer wave height, 8.0 V. Data were analysed using MassLynx 4.1, UniDec,[4] and PULSAR.[5] CCS calibrations were performed using alcohol dehydrogenase, dimer, MW 143000; concanavalin A, pentamer, MW 103000; horse heart myoglobin, MW 17600 (all Sigma).[6] CCS values were calculated using IMPACT.[4]

The repeat domain was initially modelled in a near-linear conformation and allowed to compact in two phases. The repeat domain was first placed in 0.154 M NaCl (aq) inside a

triclinic (95 Å, 95 Å, 230 Å; 90°, 90°, 60°) simulation with approximately 60 Å between periodic images. After steepest-descent minimisation followed a 10 ps NVT simulation with position restraints applied to the protein atoms. The system was then simulated in the NVT ensemble for 100 ps with position restraints applied only to the terminal C atoms, and only in the x- and y direction, leaving the protein free to initiate folding while maintaining the longest dimension aligned with the longest box vector. With restraints still applied to the termini, a 1-ns simulation was run with a semiisotropic Berendsen barostat[7] set to atmospheric pressure in order to equilibrate the pressure. A 10-ns simulation followed under the same conditions, except that the Parrinello-Rahman barostat[8] was used. After this initial round of folding, the repeat domain was re-solvated in a smaller dodecahedral box with 77.1-Å edges, reflecting a more compact conformation. Energy minimisation and subsequent equilibration steps were repeated; except there was no initial simulation done in the NVT ensemble and that the final simulation lasted for 100 ns. The Amber ff99sb-ILDN force field[9] and the tip3p water model[10] were used to describe interatomic interactions. Virtual interaction sites[11] and constrained bond lengths[12] allowed for a 4-fs time step. The v-rescale thermostat[13] was used throughout to keep the system at 300 K. All simulations were run with the GROMACS simulation package.[14]

References

1. N. Kronqvist, M. Otikovs, V. Chmyrov, G. Chen, M. Andersson, K. Nordling, M. Landreh, M. Sarr, H. Jornvall, S. Wennmalm, J. Widengren, Q. Meng, A. Rising, D. Otzen, S. D. Knight, K. Jaudzems and J. Johansson, *Nat Commun*, 2014, 5, 3254.
2. G. Askarieh, M. Hedhammar, K. Nordling, A. Saenz, C. Casals, A. Rising, J. Johansson and S. D. Knight, *Nature*, 2010, 465, 236-238.
3. R. J. Rose, E. Damoc, E. Denisov, A. Makarov and A. J. R. Heck, *Nature Methods*, 2012, 9, 1084-1086.
4. M. T. Marty, A. J. Baldwin, E. G. Marklund, G. K. Hochberg, J. L. Benesch and C. V. Robinson, *Anal Chem*, 2015, 87, 4370-4376.
5. T. M. Allison, E. Reading, I. Liko, A. J. Baldwin, A. Laganowsky and C. V. Robinson, *Nat Commun*, 2015, 6, 8551.
6. M. F. Bush, Z. Hall, K. Giles, J. Hoyes, C. V. Robinson and B. T. Ruotolo, *Anal Chem*, 2010, 82, 9557-9565.
7. H. J. C. Berendsen, J. P. M. Postma, W. F. Vangunsteren, A. Dinola and J. R. Haak, *J Chem Phys*, 1984, 81, 3684-3690.
8. M. Parrinello, *J. Appl. Phys.*, 1981, 52, 7182-7190.
9. K. Lindorff-Larsen, S. Piana, K. Palmo, P. Maragakis, J. L. Klepeis, R. O. Dror and D. E. Shaw, *Proteins*, 2010, 78, 1950-1958.
10. W. L. Jorgensen, J. Chandrasekhar, J. D. Madura, R. W. Impey and M. L. Klein, *J. Chem. Phys.* , 1983, 79, 926–935.
11. K. A. Feenstra, B. Hess and H. J. C. Berendsen, *J Comput Chem*, 1999, 20, 786-798.
12. B. Hess, *J Chem Theory Comput*, 2008, 4, 116-122.
13. G. Bussi, D. Donadio and M. Parrinello, *J. Chem. Phys.*, 2007, 126, 014101.
14. S. Pronk, S. Pall, R. Schulz, P. Larsson, P. Bjelkmar, R. Apostolov, M. R. Shirts, J. C. Smith, P. M. Kasson, D. van der Spoel, B. Hess and E. Lindahl, *Bioinformatics*, 2013, 29, 845-854.

Table S1. Theoretical and experimental CCS and observed charge state centroids for all spidroin variants. The corresponding charge states for each CCS is given in brackets. CCS values for crystal structures were calculated using the PA method with the scaling factor derived by Bush *et al.* (Anal. Chem 2012, 82:9557-65).

Protein	MW (Da)	Calc CCS	Exp CCS	Avg. charge
NT Monomer				
pdb ID 3LR2	13864.5	1410 Å^2	1471 Å^2 (7+)	6.45
pdb ID 2LPJ		1603 Å^2		
NT Dimer				
pdb ID 3LR2	27729.0	2390 Å^2	2310 Å^2 (9+)	8.91
CT Dimer				
pdb ID 3MFZ	22897.2	2234 Å^2	2365 Å^2 (9+)	8.78
NT2repCT				
Monomer	33278.4	-	2810 Å^2 (12+)	12.47
Dimer	66556.8	-	3979 Å^2 (15+)	14.71
Tetramer	133113.6	-	6730 Å^2 (24+)	23.50

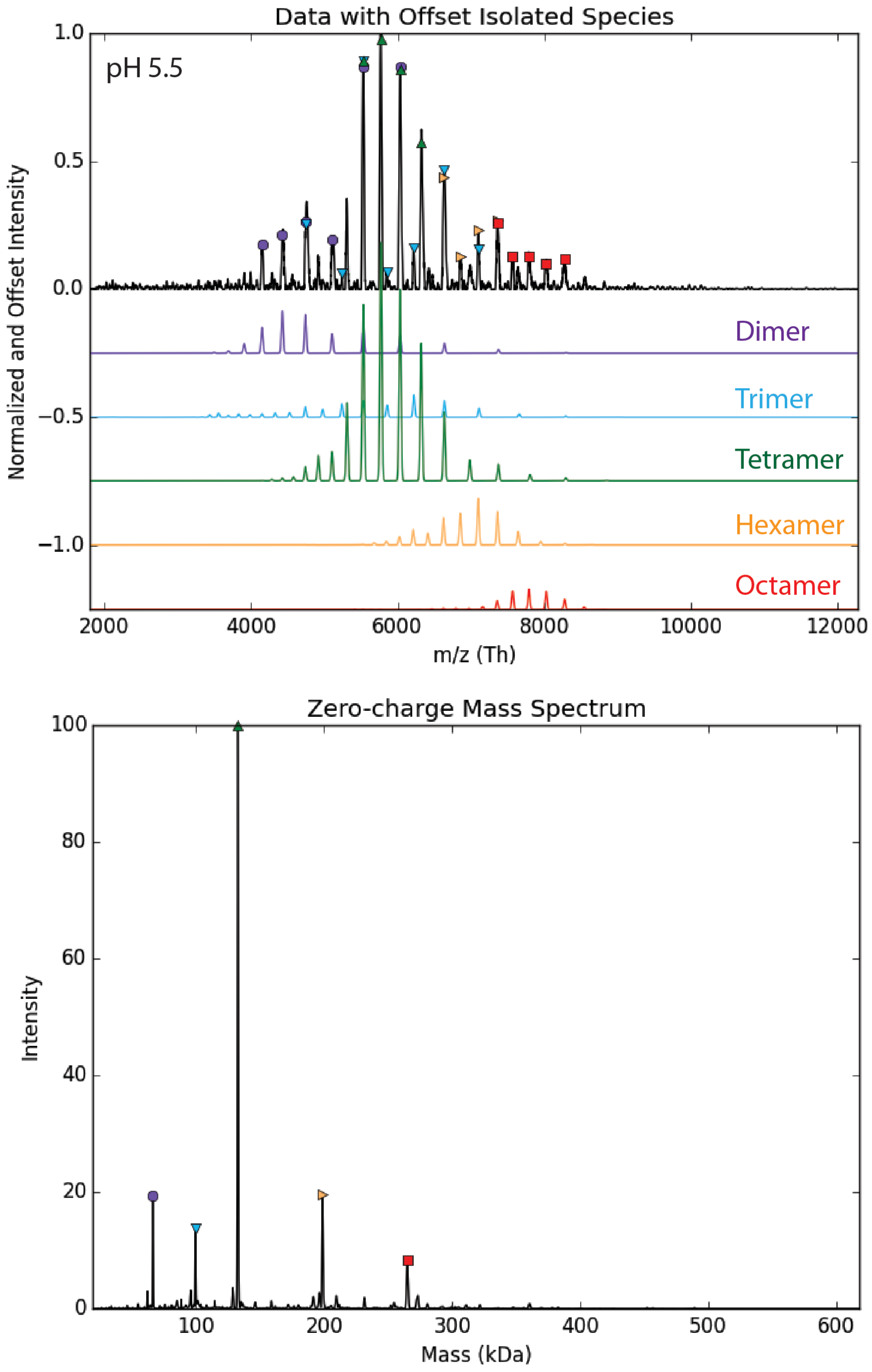

Figure S1. Deconvolution of NT2repCT spectra at pH 5.5 shows the presence of higher oligomers composed of up to 8 NT2repCT molecules. Low-intensity peaks corresponding to decamers were also observed, but no charge state envelope information could be extracted. Figures were generated using the UniDec software.

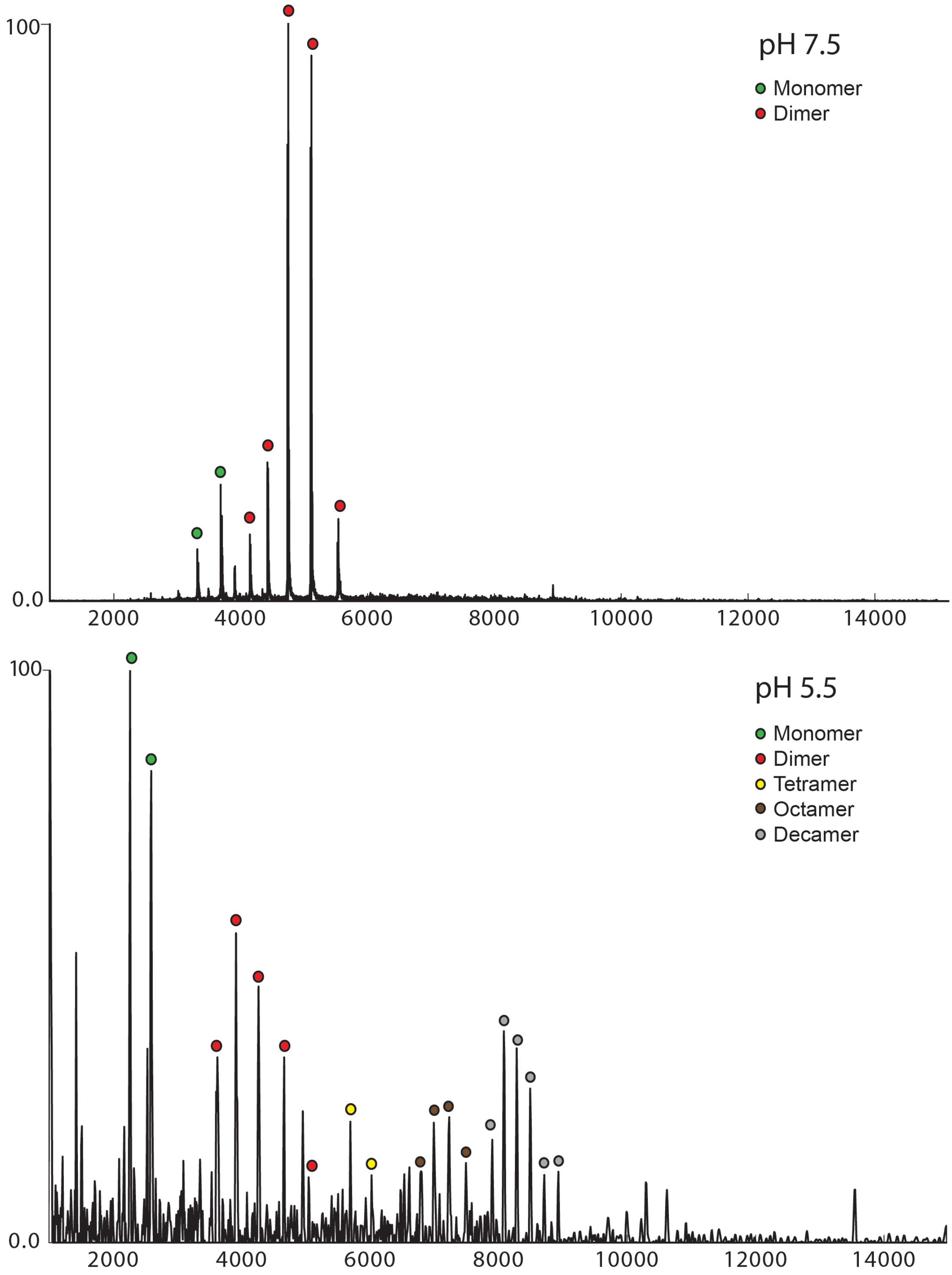

Figure S2. Mass spectra of NT2repCT at pH 7.5 and 5.5 recorded on a modified Q-Exactive Orbitrap mass spectrometer. At pH 5.5, fragmentation of the spidroins at an HCD cell voltage of 200 V shows the presence oligomers of up to ten NT2repCT molecules.

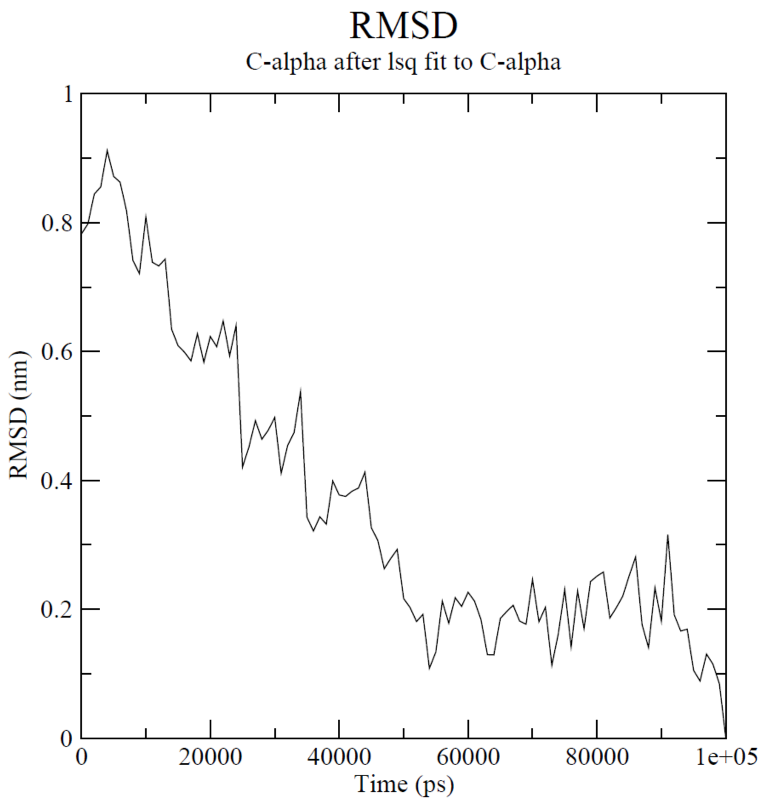

Figure S3. The linker adopts a compact conformation during MD simulations. Starting from an extended conformation, the linker rapidly collapses during all-atom MD simulations in water. The preference for the compact structure is indicated by the converging RMSD values after >50 ns. The RMSDs are calculated relative to the structure in the last frame of the trajectory.
